# Drivers’ Subjective Assessment of the Ease of Finding a Vacant Parking Space in an Area Equipped with Vehicle Detection Devices

**DOI:** 10.3390/s22186734

**Published:** 2022-09-06

**Authors:** Agata Kurek, Elżbieta Macioszek

**Affiliations:** Department of Transport Systems, Traffic Engineering and Logistics, Faculty of Transport and Aviation Engineering, Silesian University of Technology, Krasińskiego 8 Street, 40-019 Katowice, Poland

**Keywords:** magnetic sensors, dynamic parking information, paid parking zone, logit model

## Abstract

The growing traffic on city streets leads to traffic disruptions, lowering the level of road safety, as well as the problem of finding a vacant parking space. Drivers looking for a vacant parking space on the street generate so-called search traffic. Paid parking zones are introduced to increase the availability of parking spaces for more drivers in many cities around the world. The development in the technology and information sector has contributed to the development of systems guiding drivers to vacant parking spaces. This article aims to analyze drivers’ subjective assessment of the ease of finding a vacant parking space in an area equipped with vehicle detection devices. Data from the Municipal Roads Authority in Gliwice (Poland) were obtained for the study, covering the use of parking spaces in the paid parking zone covered by dynamic parking information. Moreover, a survey was conducted among users of the paid parking zone in Gliwice. The answers of the respondents were used to build a logit model that allows determining the probability of a driver’s positive subjective assessment of the ease of finding a vacant parking space in an area equipped with vehicle detection devices. The results from the model allow the characterization of drivers who positively assess the ease of finding a vacant parking space in the area equipped with vehicle detection devices. In addition, it is possible to reach a group of drivers who negatively assessed the ease of finding a vacant parking space to learn about the factors that may cause them to change their assessment to a positive one. The research results allow city authorities to better manage parking spaces equipped with vehicle detection devices in the paid parking zone. This may change the negative assessment of the ease of finding a vacant parking space into a positive one.

## 1. Introduction

The growing use of cars in travel causes traffic disruptions, lowering the level of road safety and increasing the negative impact of transport on the environment. The lack of space in built-up areas makes it impossible to expand road infrastructure. Moreover, increasing the space for cars is not in line with the sustainable development of transport and the idea of a smart city [1,2,3,4]. Scientists and engineers in the world conduct research and look for solutions aimed at the effective use of the already existing transport infrastructure [5]. New technologies and systems allow the use of innovative solutions in the area of transport.

The lack of or presence of a small number of parking spaces means that drivers circle around their destination looking for vacant parking spaces, which leads to so-called search traffic [6,7,8,9]. Paid parking zones are being introduced in areas with multiple destinations and a shortage of parking in many cities around the world. As a result, the availability of parking spaces for a larger number of drivers is increased [10]. Research conducted in the field of the impact of introducing parking fees and increasing their amount shows that drivers change their traffic behavior by choosing, e.g., shorter parking times [11,12] or any other means of travel [13,14]. Therefore, an appropriate parking fee can be a tool to reduce the share of car transport in urban areas [15]. The problem of vacant parking spaces is also caused by their uneven use in time and space. The use of parking spaces varies according to time and space as well as the volume of road traffic. The values are also influenced by random events such as road accidents, road works, events organized in the city, etc. [16,17]. Therefore, the amount of parking fees in cities around the world differs according to the area in which they are located. In recent years, research has also been carried out on the dynamic determination of the parking fees in paid parking spaces [10].

Drivers looking for a vacant parking space in areas with high parking needs feel frustrated about circling close to their travel destination. Providing drivers with information about the availability of parking spaces makes it easier for them to find a vacant parking space and shortens the time of searching for it [18]. In the case of drivers who do not know the area where the destination is located, informing them about vacant parking spaces also provides them with information about the area where they should be looked for. Drivers looking for a vacant parking space and aware of the locations of vacant parking spaces do not generate low-speed traffic, which disrupts the traffic conditions of other road users. Additionally, there are no situations of sudden braking when the driver notices a vacant parking space at the last moment, which may lead to the occurrence of a road incident.

This article aims to analyze drivers’ subjective assessment of the ease of finding a vacant parking space in an area equipped with vehicle detection devices. The research questions reported by the authors before conducting the research were as follows: What factors influenced the drivers’ subjective assessment of the ease of finding a vacant parking space in an area equipped with vehicle detection devices? Which group of drivers should be reached to learn about the factors that may cause them to change the negative subjective assessment of the ease of finding a vacant parking space in an area equipped with vehicle detection devices? The research results allow the characterization of drivers who positively assess the ease of finding a vacant parking space in an area equipped with vehicle detection devices. In addition, it is possible to reach a group of drivers who negatively assessed the ease of finding a vacant parking space to learn about the factors that may cause them to change their assessment to a positive one. The research results allow city authorities to better manage parking spaces equipped with vehicle detection devices in the paid parking zone. This may change the negative assessment of the ease of finding a vacant parking space into a positive one. According to the authors’ knowledge, no studies of the factors influencing the drivers’ subjective assessment of the ease of finding a vacant parking space in an area equipped with vehicle detection devices were conducted in Poland. Data from the Municipal Roads Authority in Gliwice, covering the use of parking spaces in the paid parking zone covered by dynamic parking information, were obtained for the research. Moreover, a survey was conducted among users of the paid parking zone in Gliwice. The article consists of 6 parts. In the second part, after the introduction, a literature review is presented in the field of various techniques used to collect data on the use of parking spaces and systems guiding drivers to vacant parking spaces. Materials and methods are presented in the next section of the article. The research area and methods of data collection, correction, and analysis were characterized. The fourth chapter covers the analysis of the use of parking spaces and the rotation indicator in 2019, as well as the responses of the respondents. The fifth chapter presents the analysis of the correlation of variables obtained from the survey data. Then, the constructed logit model was presented, which allows for determining the probability of a driver’s positive subjective assessment of the ease of finding a vacant parking space in an area equipped with vehicle detection devices. The article ends with a discussion and conclusions from the research and analysis carried out.

## 2. Literature Review

The development in the technology and IT sector allows for the wide implementation of innovations in the field of transport. Engineers and scientists around the world conduct research and look for solutions that can contribute to increasing the efficiency of the existing transport infrastructure. Authors A. Fahim et al. [19] presented an overview of several smart parking systems solutions described in the literature around the world. The advantages and disadvantages of particular detection devices which are used in parking are presented. This literature review focuses on the presentation of 3 groups of solutions for monitoring the use of parking spaces: processing technique and video detection, sensors, Global System for Mobile (GSM), and radio-frequency identification (RFID). These groups were selected because of their most common use in the area of parking. One of them is the image processing technique and video detection, which are used in many areas [20,21,22,23]. Sensors mounted in surfaces have also been used to recognize the occupancy of parking spaces [24,25,26]. The Global System for Mobile (GSM) and radio-frequency identification (RFID) systems have also been used in parking areas [27,28,29]. The use of these systems is aimed at making it easier for drivers to find a vacant parking space [30,31], dynamic pricing for parking [15,32], allowing drivers to book a vacant parking space [33,34], or forecasting the use of parking spaces for better management [35,36,37].

The first group of works relating to the use of image processing to analyze the use of parking spaces is [38]. The paper presented the project Intelligent Parking Space Detection System Based on Image Processing, consisting of 5 modules, which allow the collection of data on the use of parking spaces and provide information about vacant parking spaces to drivers on information boards. N. True [39] also used an image processing technique to detect vacant parking spaces. This paper presented a computer vision algorithm that uses a combination of car feature point detection and color classification to detect vacant parking spaces. The work [40] presented the use of image recognition using deep convolutional neural networks (CNN) and the support-vector machine (SVM) binary classifier for parking space detection. For the study of the use of parking spaces using the image processing technique, video recordings from a drone have also been used, e.g., [41]. Four features were distinguished from the parking where the research was carried out: a histogram of oriented gradients, the density of the corners inside the space, the color space separating the vehicles from the background, and a binary map of each parking space. All of these parking space features were integrated to form a vector that is classified based on the support-vector machine (SVM) classifier. The work [42] presents the use of a thermal imaging camera and the implemented pre-trained vehicle detection algorithms, histogram of oriented gradient detectors, faster regional convolutional neural network (FRCNN), and modified faster RCNN deep learning networks.

In the group of works discussing the use of magnetic sensors is [43]. Authors J. Wolff et al. presented a developed parking monitoring system based on passive magnetic field sensors. The detectors can be mounted in various places, i.e., on the surface of the parking space, under the ceiling, on the sides of the parking space, etc. The test results show that in the case of placing the sensors in the pavement, in most cases, the signal is not disturbed by vehicles parked in neighboring places, which is the case when the sensors are mounted on the wall. Authors V. W. Tang et al. [44] presented an intelligent parking management system using wireless sensor networks. This system is divided into three parts: wireless sensor, database, and application. The application system is responsible for monitoring and detecting the use of parking spaces, collecting parking fees, detecting illegal parking, and generating statistics and reports to analyze the operation of the parking system. The use of WSN was also presented by the authors. The work [45] presented the use of the ZigBee network to guide drivers to vacant parking spaces. A vehicle entering parking equipped with ZigBee devices is recognized by the ZigBee coordinator. Information about vacant parking spaces is sent to the ZigBee coordinator. Then, a vacant parking space is reserved using the acknowledgment package (ACK). The work [46] presented an algorithm using broadcasting techniques, a solution informing drivers about the situation in parking in urban traffic conditions. The information provided to vehicles by parking meters was divided into atomic and aggregated information. The first is information from one parking meter, and the second is the aggregate information about the area that includes more than one parking meter. The proposed algorithm can be used to send aggregated information to further regions and to limit atomic information to local areas. The authors R. Lu et al. [47] proposed the vehicular ad hoc network (VANET)-based smart parking (SPARK) scheme. The presented diagram of intelligent parking guides drivers to vacant parking spaces, provides intelligent protection against theft, and enables the dissemination of parking information. Thanks to the intelligent parking system proposed by the authors, the time delay in searching for a vacant parking space is low.

The last group of works includes GSM and RFID systems, which allow for controlling the use of parking, but without accuracy to a single parking space, only to the area. The use of GSM in the management of better use of parking spaces was presented by the authors G. Zhang et al. [48]. The article presents a parking space management system based on the programmable system-on-chip (PSoC). This system collects information about available parking spaces via the GSM network. Drivers can find a vacant parking space by sending an inquiry or ordering a message via SMS or the Internet. According to the authors, this system can solve the problems related to the growing demand for parking spaces and insufficient land for the construction of new parking. Authors E. Karbab et al. [49] presented a smart parking platform that uses wireless sensor networks (WSN) and radio frequency identification (RFID) technology. The proposed platform ensures smooth integration of RFID and WSN, along with the grouping of sensors with a single signal into several points. The results show that the proposed system lowers costs and also energy consumption.

The results of the research presented in the above-mentioned studies indicate a very good level of recognition of the occupancy of parking spaces in various systems and the possibility of shortening the time of searching for a parking space by drivers, thanks to the use of tools to guide them to vacant parking spaces. Drivers do not waste time looking for vacant parking spaces. In addition, the traffic generated by people looking for a parking space is reduced, and the likelihood of sudden braking by the driver who noticed a vacant parking space at the last moment is reduced.

## 3. Material and Methods

The city of Gliwice, located in the southern part of Poland, was selected as the research area. In 2021, the population density in Gliwice was 1322 people/1 km^2^, and the motorization index-715.2 passengers cars/1000 inhabitants. According to [50], 18.53% of the area of Gliwice is occupied by service and residential areas, 8.15% is occupied by service, industrial, and production areas, 8.20% is occupied by railway areas, basic road, and air communication systems, 58.90% is occupied by areas of greenery and surface waters, and the remaining areas cover 6.22% of the city’s area. One of the most modern intelligent traffic control systems was implemented in Gliwice in the years 2007–2013 as part of the project “Expansion of the detection system in the city of Gliwice along with the modernization of selected traffic lights, stage I”, being the first of its kind in the area of the Silesian Voivodeship. The applied solutions allow for traffic control in specific road conditions. The devices that make up the system collect data from various sources, including presence and speed detectors at intersections with traffic lights and on traffic routes, parking space occupancy detectors, public transport vehicle location systems, along with information about the delay, and metrology stations. A department of the Municipal Roads Authority-Traffic Control Center was also launched as part of the system implementation. The employees of the Traffic Control Center react in real time with the support of the system to the current situation on the roads to maximize traffic flow and maintain high safety [51,52]. Therefore, the city of Gliwice was selected as the case study.

According to the regulations in Poland, paid parking zones can be introduced in areas with a shortage of parking spaces. The purpose of introducing PPZ is to increase the rotation of parking vehicles or to implement the local transport policy, in particular, to limit the accessibility of this area for motor vehicles or to introduce preferences for public transport. The fee for parking vehicles in the paid parking zone may be collected in a designated place, on specific working days, at certain hours, or 24 h a day [53].

**Figure 1:**
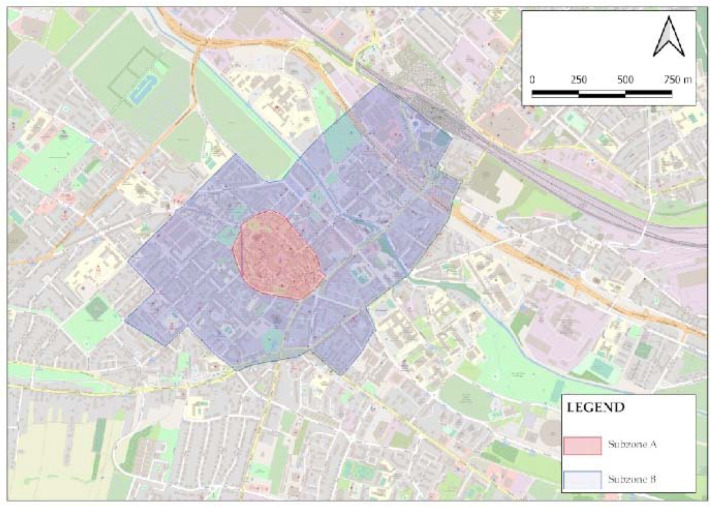
The paid parking zone in Gliwice divided into subzones.

The paid parking zone (PPZ) has been operating in the city of Gliwice since 2012, which is divided into Subzone A (29 streets) and Subzone B (60 streets) (Figure 1) [54]. The market is located in the area designated by Subzone A of the PPZ. On the market and around it are mainly restaurants, cafes, commercial and service buildings, etc. In turn, in the area of subzone B, there are mainly commercial and service buildings, the City Hall, the Silesian University of Technology, etc. The area covered by the PPZ is the very center of the city, with a large number of traffic generators

In the paid parking zone, the parking fee is collected from Monday to Friday from 09:00 to 17:00. The fee for parking in subzone A is higher than in subzone B [54]:

- First hour: Subzone A-3.00 PLN (~0.65 €); Subzone B-2.00 PLN (~0.43 €);
- Second hour: Subzone A-3.50 PLN (~0.76 €); Subzone B-2.40 PLN (~0.52 €);
- Third hour: Subzone A-4.20 PLN (~0.91 €); Subzone B-2.80 PLN (~0.61 €);
- Fourth and the next hour: Subzone A-3.00 PLN (~0.65 €); Subzone B-2.00 PLN (~0.43 €);
- Whole day: Subzone A: 25.00 PLN (~5.42 €); Subzone B: 12.00 PLN (~2.60 €).

PPZ users can pay a fee for parking at the parking meter and via the mobile application. Registered residents of an estate located on the streets where PPZ is designated are entitled to a lump sum of 120 PLN (~25 €) per year, called the “Resident’s Subscription,” for parking only one car. If the fee for parking in the PPZ is not paid, an additional fee of 20 PLN (~4 €) or 50 PLN (~10 €) is charged, depending on the date of its payment. The fee for parking in the PPZ is not required from:

- Disabled people with a parking card,
- Those who drive one-wheeled vehicles,
- Taxis at their designated parking spaces,
- Public transport vehicles stopping at stops,
- Vehicles of the Municipal Police in Gliwice,
- Permanently marked vehicles of the PPZ operator during the performance of the duties of the PPZ operator.

**Figure 2:**
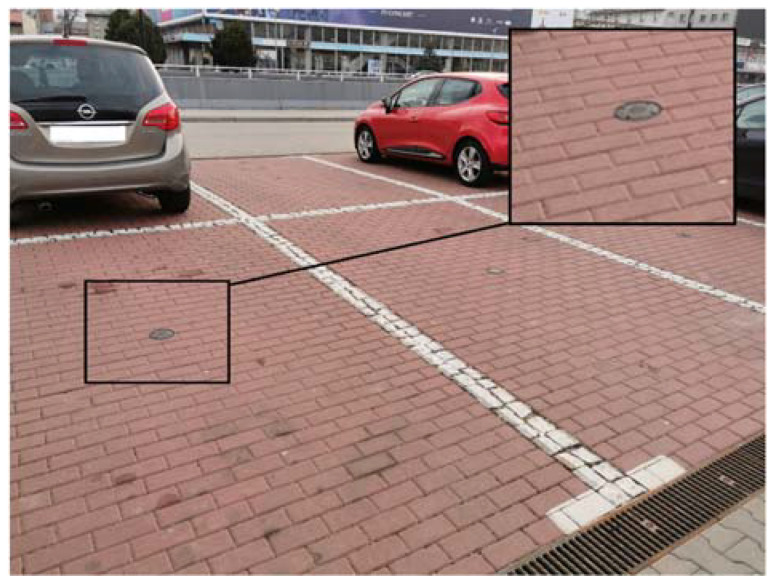
Parking spaces are equipped with a magnetic sensor in PPZ in Gliwice.

**Figure 3:**
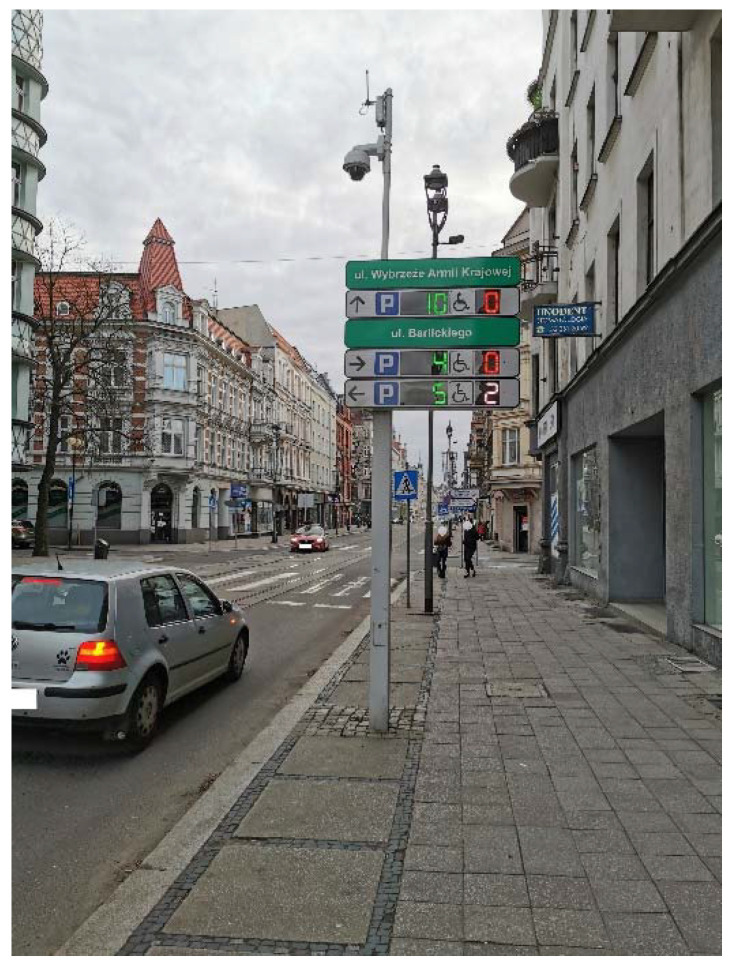
Dynamic parking information board in PPZ covered by DPI in Gliwice.

Magnetic sensors were installed in 751 parking spaces in their surfaces to detect the presence of a vehicle (Figure 2—a black circular object in the center of a parking space). A vehicle detection system in parking spaces allows for the monitoring of the use of parking spaces in real time and makes it easier for drivers to search for vacant parking spaces. The magnetic sensor, based on the continuous measurement of the Earth’s magnetic field, detects the vehicle by disturbing the magnetic field generated by the induction loop. Then, several concentrators located within the paid parking zone collect data from sensors. Through a fiber-optic network, data from the concentrators are sent to the controller, which displays information about vacant parking spaces on the dynamic parking information board (Figure 3). The dynamic parking information board allows providing information about available parking spaces to the driver so that he can make decisions about the area where he wants to park. The place of installation of the dynamic parking information board should be the transit road in the parking, from which there are streets with parking spaces. A driver going through the transit road sees several dynamic parking information boards from a distance and, based on their indications, can decide to choose an area with parking spaces. In addition, in 2019, the ITS Gliwice application was launched, in which drivers are provided with information about the number of vacant parking spaces in a given area.

Data on the use of parking spaces in the paid parking zone covered by dynamic parking information were obtained from the Municipal Roads Authority in Gliwice. The data included the number of parked vehicles in each hour of 2019. First of all, data analysis included the verification of gaps and incorrect data.

Data collection from vehicle detection systems is associated with the occurrence of inaccuracies or missing data. This is caused by random system failures, e.g., interruptions in data communication, interruptions in power supply, unfavorable weather conditions, etc. In the literature, papers can be found discussing methods of supplementing incorrect and missing data [55,56]. The missing values are random in the data set in the case of the analyzed data from parking space occupancy sensors. Missing data are independent of variables in the dataset. Such missing data are called missing completely at random (MCAR) [57], which can be replaced with an average value according to Formula (1):

(1) $$Y_{B}=\frac{\sum_{i=1}^{n_{z}}Y_{Z_{i}}}{n_{Z}}$$

where:

$Y_{B}$—missing value,
$Y_{Z_{i}}$—known values,
$n_{Z}$—the number of known values in the entire sample *n*.

An analysis of the variability in the use of parking space and the rotation indicator in each hour of 2019 in particular months was carried out for the prepared data. The use of the parking space is the number of parking spaces occupied by vehicles per unit time, expressed by Formula (2) [58]:

(2) $$u_{p}=\frac{V_{p}}{S_{p}}\cdot 100\;\left[\%\right]$$

where:

*u_p_*—use of the parking space (%);
*V_p_*—number of parked vehicles in the analyzed time unit (veh.);
*S_p_*—number of parking spaces in the analyzed area (parking space).

The rotation indicator is the average number of parked vehicles that use one parking space in a given period of the analysis expressed by Formula (3) [58]:

(3) $$r_{p}=\frac{V_{ps}}{S_{p}}\;\left[\frac{veh.}{parking\;space}\right]$$

where:

*r_p_*—rotation indicator (veh./parking space);
*V_ps_*—the number of vehicles using a particular parking space in the analyzed time unit (veh.);
*S_p_*—number of parking spaces in the analyzed area (parking space).

A survey of users of the Paid Parking Zone in Gliwice was also conducted. A total of 7184 drivers used the Paid Parking Zone on 16 October 2019 (Wednesday). Based on Formula (4), the minimum sample size for the survey was determined:

(4) $$N_{min}=\frac{N_{p}\cdot \left(\alpha^{2}\cdot f\cdot \left(1-f\right)\right)}{N_{p}\cdot e^{2}+\alpha^{2}\cdot f\cdot \left(1-f\right)}$$

$$N_{min}=365$$

where:

*N_min_*—minimum sample size;
*N_P_*—the size of the sampled population;
*α*—confidence level for the results, the value of the Z result in the normal distribution for the assumed significance level, 1.96;
*f*—fraction size, 0.5;
*e*—assumed maximum error, 5%.

A total of 1356 responses were collected from the respondents. The results of the questionnaire survey were checked for completeness of the answers. Responses containing “I do not know”, “I refuse to answer”, or “I have no opinion” were rejected. A total of 1198 respondents’ answers were used for the analysis. The following information about the respondents was collected:

- Gender;
- Age;
- Place of residence;
- Professional status;
- Monthly income (net) (PLN);
- Frequency of using the car as a driver;
- The number of kilometers driven during a year;
- The number of years of having a driver’s license;
- The number of cars in the household;
- Trip purpose;
- Frequency of using PPZ in Gliwice;
- Subzone where respondents parked;
- The reason for choosing a parking space;
- Estimated parking time;
- Method of payment for parking;
- Guidance tools used to find a vacant parking space;
- Drivers’ subjective assessment of the ease of finding a vacant parking space in an area equipped with vehicle detection devices;
- Subjective assessment of the search time for a vacant parking space;
- The number of passengers (apart from the driver) traveling by car.

## 4. Analysis of Drivers’ Behavior

### 4.1. Analysis of the Use of Parking Space and Rotation Indicator

**Figure 4:**
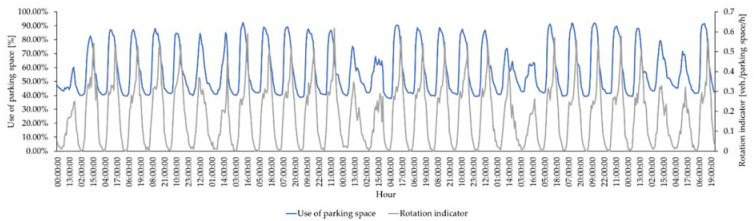
Use of parking space and rotation indicator in PPZ covered by DPI in September 2019.

This part of the article presents the results of the analysis of the use of parking spaces and the rotation indicator in parking spaces in the paid parking zone equipped with vehicle detection devices. The analysis covered every hour in every month of 2019. Figure 4 presents the use of parking space and rotation indicators in the PPZ covered by DPI in September 2019 to illustrate the variability of the value of the use of the parking space and the rotation indicator. The use of the parking space and the rotation indicator are characterized by similar values on working days in all months in 2019. A decrease in these values is noticeable on weekend days compared to working days. The use of parking space and the rotation ratio on Saturdays are higher than on Sundays. The highest values of the use of parking spaces were in October (96.76%), and the lowest values were in August (86.11%). The highest values of the rotation indicator were obtained in March, April, and December (respectively, 0.64, 0.64, and 0.63 vehicles/parking space/h), while the lowest were in January, February, October, and November (respectively, 0.53, 0.57, 0.57 and 0.56 vehicles/parking space/h). During working days, the highest use of the parking space occurs between 08:00 and 17:00, and the lowest use occurs in the morning and night hours. The rotation indicator on working days is the highest between 13:00 and 18:00 and between 07:00 and 13:00. It is lower between 19:00 and 20:00, and at night, it is the lowest.

The values of the use of parking space and the rotation indicator on non-working days are lower than on working days and on the days immediately before and after them. Table 1 presents a list of days on which a decrease in value is noticeable to the corresponding days in particular months of 2019, along with information about public holidays occurring on those days. In February, March, July, September, and October, there are no days when these values can deviate. There was no public holiday during these months. On 1–2 June 2019 (Saturday and Sunday), the values of the use of parking space and the rotation indicator are higher than the average values for Saturdays and Sundays in June 2019. The increase in these values on June 1 may be related to the occurrence of a child’s day on that day in Poland. In addition, on 1–15 December 2019, the use of parking spaces was not less than 50%. However, on 16 December 2019, in the night and morning hours, this value drops below 50%.

### 4.2. Survey Data Analysis

**Figure 5:**
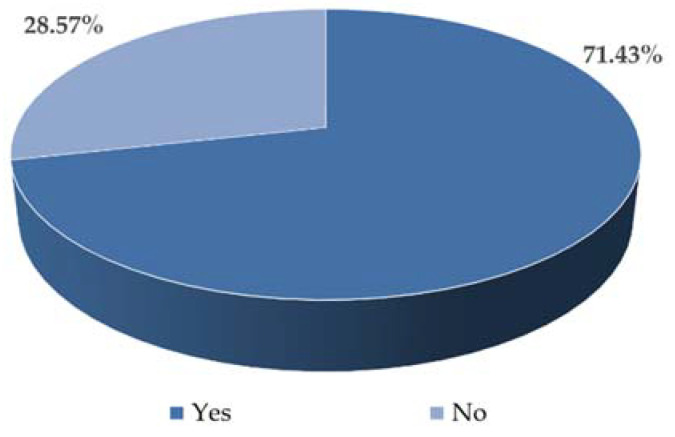
Distribution of respondents’ answers regarding the ease of finding a vacant parking space in an area equipped with vehicle detection devices.

This chapter presents the results of the surveys. Figure 5 shows the distribution of respondents’ answers regarding the ease of finding a vacant parking space in the paid parking zone in Gliwice. Over 71% of respondents answered that finding a vacant parking space did not make it difficult for them to find a vacant space to leave the vehicle. In turn, over 28% of the respondents answered that they had a problem with finding a vacant parking space.

Table 2 shows the respondents’ answers to the questions included in the survey according to the respondents’ answers regarding the ease of finding a vacant parking space in the paid parking zone in Gliwice. Over 55% of men negatively assessed the ease of finding a vacant parking space, and over 42% of men assessed this positively. Over 57% of women said that they had no problem with finding a vacant parking space, and 44% of women had a problem with finding a vacant parking space. People aged 18–39 mostly (over 66% of respondents) assessed finding a vacant parking space as easy, and more than 62% of people in this age group assessed it as difficult. Over 37% of people aged 39 and over assessed finding a vacant parking space as difficult, while over 33% of people of this age assessed it as easy. The inhabitants of Gliwice and the Gliwice county had a problem with finding vacant parking spaces (60% of respondents). Furthermore, 60% of people living outside Gliwice county positively assessed the ease of finding a vacant parking space. Over 75% of people working or studying said that finding a vacant parking space was not difficult for them, while more than 66% of people with such a professional status had a problem with finding a vacant parking space. The group of people with a different professional status included the unemployed, retirees, pensioners, and self-employed. Over 33% of people with a different professional status answered that they had a problem with finding a vacant parking space, while over 24% of these people positively assessed the ease of finding a vacant parking space. The respondents whose monthly (net) income is 0–3 499 PLN (~0–717 €) mostly negatively assessed the ease of finding a vacant parking space (62.5%), whereas over 56% of respondents assessed it positively. Almost 44% of the respondents with a monthly (net) income above 3500 PLN (~718 €) positively assessed the ease of finding a vacant parking space, while 37.5% of respondents assessed it negatively. People who traveled by car more often (3–7 times a week) negatively assessed the ease of finding a vacant parking space (over 77% of respondents), and over 57% of people answered that they had no problem with finding a vacant parking space. More than 42% of respondents traveling by car less than 3–7 days a week positively assessed the ease of finding a vacant parking space, whereas over 22% of these people had a problem with finding a vacant parking space. People traveling by car more than 10,000 km per year more often said that they had a problem with finding a vacant parking space (over 66% of respondents), and 52% of respondents traveling less than 10,000 km per year by car had no problem with finding a vacant parking space. Over 42% of respondents who have a 1–9-year-old driving license positively assessed the ease of finding a vacant parking space, whereas over 33% of respondents assessed it negatively. More than 66% of people who have a longer driving license (10 years or more) answered that they had a problem with finding a vacant parking space, and over 66% of people did not have a problem with it. For people who have only one car in their household, the majority (50% of respondents) answered that they had a problem with finding a vacant parking space, whereas over 35% of these people had no problem with it. Over 64% of respondents who have 2 or more cars in their households positively assessed the ease of finding a vacant parking space, whereas over 64.44% of respondents assessed it negatively. The majority of respondents whose purpose of travel was work or school (over 22% of respondents) assessed finding a vacant parking space as easy, and over 15% of respondents assessed it as difficult. Over 84% of respondents whose purpose was not working or school (shopping, healthcare, family gathering, social gathering, official matters, etc.) assessed finding a vacant parking space as easy, and over 77% of respondents assessed it as difficult. Most drivers using PPZ in Gliwice 1–7 times a week (over 61% of respondents) stated that they had a problem with finding a vacant parking space, whereas 40% of respondents had no problem with it. Furthermore, 60% of drivers who less frequently use the PPZ in Gliwice positively assessed finding a vacant parking space, and less than 39% of respondents assessed it negatively. Over 83% of the respondents who parked in subzone A had a problem with finding a vacant parking space, whereas over 77% of respondents did not have a problem with it. Over 22% of drivers who parked in subzone B had no problem with finding a vacant parking space, whereas over 16% of respondents had a problem with it. People for whom the reason for choosing a parking space was close to their destination mostly (over 86.67% of respondents) had no problem with finding a vacant parking space, and over 38% of respondents had a problem with it. The respondents who indicated a low fee as the reason for choosing a parking space mostly (over 61% of respondents) negatively assessed the ease of finding a vacant parking space, whereas over 13% of respondents assessed it positively. The majority of drivers (over 91% of respondents) who foresee a 0–2 h parking answered that they had a problem with finding a vacant parking space, and over 67% of respondents had no problem with it. Over 32% of drivers who foresee parking for more than 2 h positively assessed the ease of finding a vacant parking space, and over 8% of respondents assessed it negatively. The method of payment by the respondents does not affect the assessment of the ease of finding vacant parking spaces (the difference is approximately 1% of respondents). Over 66% of people who do not use tools that guide them to vacant parking spaces (dynamic parking information boards, ITS Gliwice application) stated that they had a problem with finding a vacant parking space. Drivers who use dynamic parking information or the ITS Gliwice application mostly (over 55% of respondents) positively assessed the ease of finding a vacant parking space. Of the respondents who estimated the time to search a vacant parking space as 0–2 min, the majority (over 93% of respondents) answered that they had no problem with it. More than 66% of respondents who estimated the time to search for a vacant parking space to be more than 2 min negatively assessed the ease of finding a vacant parking space. Over 77% of people who traveled alone negatively assessed the ease of finding a vacant parking space, and it was positively assessed by over 73% of respondents. Drivers who traveled with passengers mostly (over 26% of respondents) said they had no problem finding a vacant parking space, and over 22% of respondents had a problem with it.

## 5. Statistical Model Development

Studies of drivers’ communication behavior are conducted with the use of various modeling techniques [59,60,61]. It is possible to use the binomial logit model for modeling a categorical variable that takes two states [62,63]. The research aimed to analyze drivers’ subjective assessment of the ease of finding a vacant parking space in an area equipped with vehicle detection devices and what is possible through getting to know the factors influencing drivers’ subjective assessment of the ease of finding a vacant parking space in an area equipped with vehicle detection devices. The driver can positively or negatively assess the ease of finding a vacant parking space in an area equipped with vehicle detection devices. Therefore, the variable Y can assume two states.

In this study, a binomial logit model was developed that presents the features influencing drivers’ subjective assessment of the ease of finding a vacant parking space in an area equipped with vehicle detection devices. The binomial logit model allows determining the probability of a phenomenon. The built logit model, based on the data presented in this article, allows learning about the factors causing an increase in the positive probability of drivers’ subjective assessment of the ease of finding a vacant parking space in an area equipped with vehicle detection devices. This model is expressed by Formula (5):

(5) $$Y=\{\begin{array}{l} 1\to \;\;\;\;\;\;\;\;\;\;\;\;\;\;\;\;\;\;\;\;\;\;\;\;\;\;the\;phenomenon\;occurs\\ 0\to \;\;\;\;\;\;\;\;the\;phenomenon\;does\;not\;occur \end{array}$$

where:

*Y*—dependent variable.

The logit model for the dependent variable *Y* is presented by Formula (6):

(6) $$p=P\left(Y=1|X_{1},\;\ldots ,\;X_{n}\right)=\frac{\exp\left(\gamma_{0}+\sum_{i=1}^{n}\gamma_{i}\cdot X_{i}\right)}{1+\exp\left(\gamma_{0}+\sum_{i=1}^{n}\gamma_{i}\cdot X_{i}\right)}$$

where:

*p*—the probability of the phenomenon occurring;
*X_i_*—independent variable (*i* = 1, …, n);
*γ*_0_—free expression;
*γ_i_*—independent variable coefficient *i*.

Model (6) is transformed by the logarithm into the odds ratio (the ratio of the probability of a phenomenon occurring to the probability that the phenomenon will not occur), expressed by Formula (7):

(7) $$logit\left(p\right)=ln\frac{p}{1-p}=\gamma_{0}+\gamma_{1}\cdot X_{1}+\gamma_{2}\cdot X_{2}+\ldots +\gamma_{n}\cdot X_{n}$$

The responses of the respondents were adopted as variables for the model:

*y*—drivers’ subjective assessment of the ease of finding a vacant parking space in an area equipped with vehicle detection devices;
*x_G_*—gender;
*x_A_*—age;
*x_R_*—the place of residence;
*x_PS_*—professional status;
*x_I_*—monthly income (net) (PLN);
*x_FUC_*—frequency of using the car as a driver;
*x_KC_*—the number of kilometers driven during a year;
*x_YDL_*—the number of years of having a driver’s license;
*x_CH_*—the number of cars in the household;
*x_TP_*—trip purpose;
*x_FUPPZ_*—frequency of using PPZ in Gliwice;
*x_S_*—subzone where respondents parked;
*x_RCPS_*—the reason for choosing a parking space;
*x_PTP_*—predicted parking time;
*x_PM_*—method of payment for parking;
*x_DPIBIGA_*—use of guidance tools that allow finding a vacant parking space;
*x_PTSPS_*—subjective assessment of the search time for a vacant parking space;
*x_P_*—the number of passengers (apart from the driver) traveling by car.

First, a correlation analysis was performed using the Statistica program. Independent variables correlated with the dependent variable are gender, age, professional status, place of residence, monthly income, number of kilometers driven during the year, the number of years of having a driver’s license, the reason for choosing a parking space, frequency of using the PPZ in Gliwice, use of guidance tools that allow finding a vacant parking space as well as a subjective assessment of the search time for a vacant parking space. The independent variables age, professional status, and the number of years of having a driver’s license were rejected due to a correlation with other independent variables that were correlated with the dependent variable. The model assumed independent variables were characterized by a strong correlation with the dependent variable and a weak correlation with each other: gender, the place of residence, monthly income, the number of kilometers driven during a year, frequency of using PPZ in Gliwice, the reason for choosing a parking space, use of guidance tools that allow finding a vacant parking space, and subjective assessment of the search time for a vacant parking space.

Table 3 presents the variables adopted for modeling with the symbols assigned to them.

Two logit models were developed. The first model includes all the variables that were adopted after the correlation analysis. The second one takes into account the variables whose *p*-values obtained in the first model were less than 0.05. Table 4 shows the results of the developed logit models.

In model 1, the greatest impact on drivers’ subjective assessment of the ease of finding a vacant parking space in an area equipped with vehicle detection devices is a subjective assessment of the search time for a vacant parking space and the use of tools to guide to vacant parking spaces. On the other hand, the gender and monthly income (net) of the respondents have no influence. The model correctly predicts 77.78% of cases of a driver’s negative subjective assessment of the ease of finding a vacant parking space in an area equipped with vehicle detection devices and 93.33% of drivers’ positive subjective assessment of the ease of finding a vacant parking space in an area equipped with vehicle detection devices. The Nagelkerke R^2^ is 0.562.

After rejecting the variables with a *p*-value greater than 0.05 in model 1, model 2 was developed with R^2^ Negelkerke equal to 0.511. R^2^ Negelkerke takes values from 0 to 1, and the closer the value is to 1, the better the fit of the model is [64]. The R^2^ Negelkerke value for model 1 is slightly higher (0.05) than the value obtained in model 2. With a slight difference in the R^2^ Negelkerke value and the fact that in model 1, the *p*-value of 2 variables takes values greater than 0.05, model 2 was considered to be a better fit. If the driver assessing the ease of finding a vacant parking space in the paid parking zone in Gliwice in parking spaces equipped with vehicle detection devices lives outside Gliwice and the Gliwice county, the probability that the driver will assess positively is 0.884 lower than in the case of respondents living in Gliwice and the Gliwice county. The greater number of kilometers traveled annually by drivers and the lower frequency of using PPZ in Gliwice by them causes a decrease in the probability of a driver’s positive subjective assessment of the ease of finding a vacant parking space in an area equipped with vehicle detection devices compared to people who drive fewer kilometers and more often use the PPZ in Gliwice (by 0.888 and 0.445, respectively). If the reason for choosing a parking space is a low price for parking, the probability of a driver’s positive subjective assessment of the ease of finding a vacant parking space in an area equipped with vehicle detection devices increases by 4.193. The respondents who do not use the tools guiding to vacant parking spaces, with a probability of 1.904 lower, positively assess the ease of finding a vacant parking space in the paid parking zone in Gliwice in parking spaces equipped with vehicle detection devices. A longer perceptible time to search for a vacant parking space reduces the probability of a positive assessment of the ease of finding it by 11.450. Table 4 presents the results of the constructed model.

Therefore, Model 2 takes the form of the Formula (8):

(8) $$y=-2.188-0.124\cdot x_{R}-0.118\cdot x_{KC}-0.809\cdot x_{FUPPZ}+1.434\cdot x_{RCPS}-0.644\cdot x_{TGVPS}-2.438\cdot x_{PTSPS}\;$$

**Figure 6:**
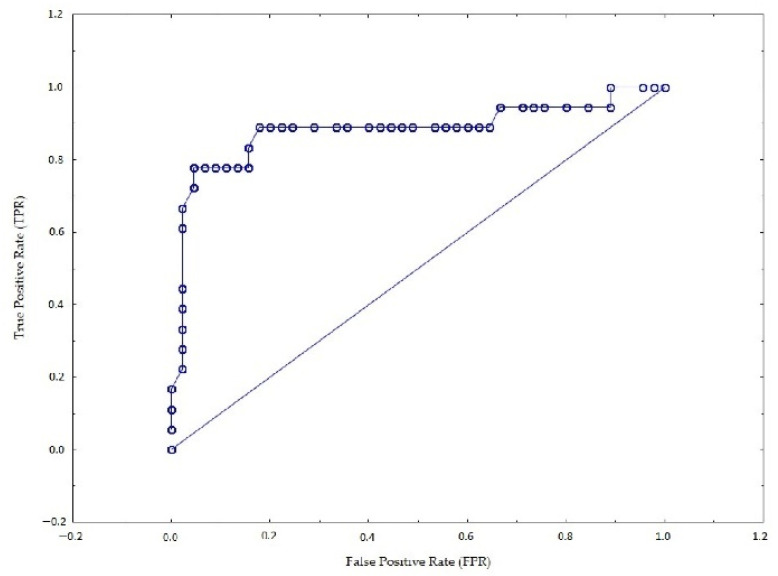
ROC curve for 1st model.

**Figure 7:**
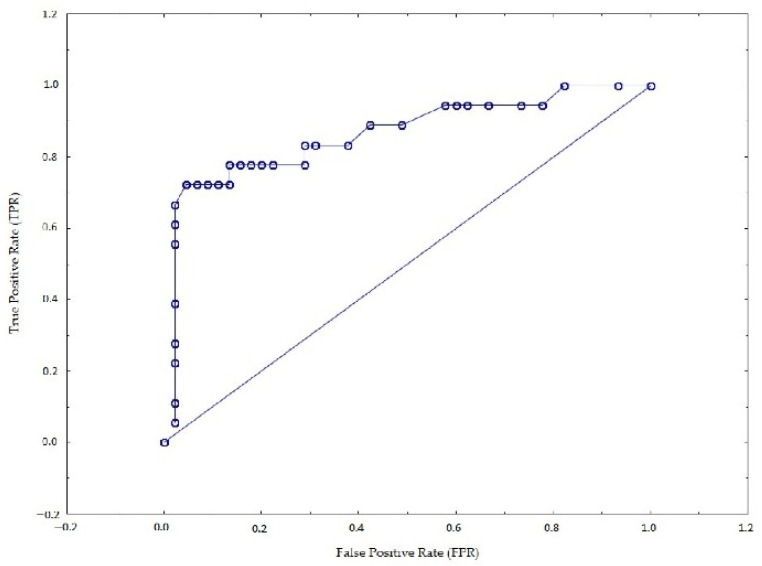
ROC curve for 2nd model.

The ROC (receiver operating characteristic) curve was determined for the developed models. The ROC curve makes it possible to visualize the sensitivity and specificity of the model. Based on the ROC curve, it is possible to find the optimal point that maximizes the value of sensitivity and specificity. The AUC (area under the curve) is calculated under the ROC curve. AUC values range from 0 to 1, and the closer the value is to 1, the better the model [64]. In the case of model 1, the area under the curve is approximately 0.88 (Figure 6), while in model 2, it is approximately 0.86 (Figure 7). The comparison of the results of both models shows that model 1 better predicts drivers’ subjective assessment of the ease of finding a vacant parking space in an area equipped with vehicle detection devices than model 2. However, with a slight difference between the AUC values for models 1 and 2 and the fact that in model 1, the *p*-value of 2 variables is greater than 0.05, model 2 was considered a better fit.

## 6. Discussion

The literature review presented in this article indicates the use of various systems to detect vehicles in parking spaces. These systems are used to guide drivers to vacant parking spaces, as well as to conduct analyses aimed at the appropriate management of parking in the city. New technologies also allow their use in the context of dynamic pricing for parking. The introduction of parking fees for parking may contribute to increasing the availability of parking spaces for more drivers. The use of guidance systems for vacant parking spaces, as well as the appropriate parking fees, contribute to the reduction of traffic generated by searching for a vacant parking space.

In all months, the use of parking space and the rotation indicator are characterized by similar values on working days (Monday–Friday). On Saturdays and Sundays, these values are lower than on working days. On Saturdays, the use of parking space and the rotation indicator take higher values than on Sundays. In October, the use of parking spaces was the highest in 2019 (the highest value was 96.76%), and the lowest use was in August (the highest value was 86.11%). The highest values of the rotation indicator can be observed in March, April, and December (the highest value was 0.64, 0.64, and 0.63 vehicles/parking space/h, respectively), and the lowest can be observed in January, February, October, and November (the highest value 0.53, 0.57, 0.57 and 0.56 vehicles/parking space/h, respectively). The greatest use of the parking space on an average working day is between 08:00 and 17:00, and the lowest is in the morning and night hours. The highest values of the rotation indicator on working days occurred between 13:00 and 18:00. The rotation indicator decreases between 07:00 and 13:00 and between 19:00 and 20:00, and the rotation indicator is the lowest during the night hours. On a public holiday, the use of parking spaces and the rotation indicator are lower than on working days, although the fee is not charged on these days. The presence of a public holiday also affects the use of parking spaces and the rotation indicator on the day before and after the public holiday. The use of parking spaces provided by the authors of J. Parmar et al. [65] ranges from 77% to 98%. The authors also note that due to the lack of information about available parking spaces, drivers have a problem with finding vacant parking spaces. In the work [66], measurements of the use of parking spaces were also carried out. The research results indicate that in November 2010, it ranged from 29% to 78%, and in February 2011, it ranged from 24% to 84%. The work [67] presents the results of the research on stopping time on parking located on the street. Based on the presented research results, the average parking time was 77 min. Parking time in parking spaces up to 1 h was 57% and over 3 h was 10%. From the research presented in [68], the authors conclude that the use of parking spaces varies depending on the time of day and the characteristics of the area. The results of the research on the use of parking spaces presented in the literature are similar to those obtained in this article.

Surveys show that women aged 18–39 living outside Gliwice and Gliwice county positively assess the ease of finding a vacant parking space. These people are working or studying and have a monthly income of over 3500 PLN (net) (~756 €). The respondents who traveled by car less than 3 times a week, drove less than 10,000 km, had a driving license for less than 10 years, and had 2 or more cars in their household had no problem with finding a vacant parking space. The aim of these people was shopping, healthcare, shopping, social gathering, family meeting, official matters, etc. They used the PPZ in Gliwice less than once a week and parked their car in subzone B. Finding a parking space was easy for people who chose a parking space because it was close to the destination, foresaw 2 h or more of parking time, paid for parking via the application, used guiding tools to find vacant parking spaces, estimated the time of looking for a vacant parking space as 0–2 min and traveled with passengers.

A presented logit model 2 that allows determining drivers’ subjective assessment of the ease of finding a vacant parking space in an area equipped with vehicle detection devices is characterized by a good fit (R^2^ Negelkerke = 0.511). It is also characterized by the ability to predict the answers “yes” = 93.33% and “no” = 77.78%. People living in Gliwice and the Gliwice county, who travel fewer kilometers annually and have a greater frequency of using the PPZ in Gliwice are more likely to positively assess the ease of finding a vacant parking space in an area equipped with vehicle detection devices than people living outside Gliwice and the Gliwice county, who travel more kilometers annually and have a lower frequency of using the PPZ in Gliwice. A driver who chose a parking space because of the low price for parking, who uses tools to guide them to vacant parking spaces, and who assesses a short search time for a vacant parking space is more likely to positively assess the ease of finding a vacant parking space in an area equipped with vehicle detection devices than the driver who chose parking because of it being close to the destination, who does not use tools to guide them to vacant parking space and who assesses long search time for a vacant parking space. The authors H. Teng et al. [69] conducted a study of factors that affect drivers’ perception of difficulties with finding a vacant parking space in New York (USA). The more time drivers have spent looking for a vacant parking space, the more likely they are to negatively assess the ease of finding a vacant parking space. Older drivers and those with a higher monthly income had less difficulty finding a parking space. In addition, the authors indicate that the amount of information about parking that the drivers had before the trip was directly related to the time of their search, which in turn influenced their perception of difficulties with parking. The simulation carried out by the authors G. Tasseron and K. Martens [70] shows that the information about vacant parking spaces and reservations does not have a large impact on the time spent looking for parking. The authors R. G. Thompson and P. Bonsall [71] also assess the influence of guiding tools on the ease of finding a vacant parking space as small. In turn, from the model presented in this article, it can be concluded that in the case of drivers parking in the PPZ covered by DPI, the use of guiding tools increases the probability of easily finding a vacant parking space. Differences in the impact of the use of guidance tools on vacant parking spaces may result from drivers’ trust in the information provided to them. The results presented in [72] show that the fact that drivers ignore information about vacant parking spaces may result from the inaccurate or outdated nature of the displayed information. It can therefore be concluded that drivers using the PPZ in Gliwice trust the guidance system to vacant parking spaces.

## 7. Conclusions

The article aimed to analyze drivers’ subjective assessment of the ease of finding a vacant parking space in an area equipped with vehicle detection devices. According to the authors’ knowledge, no studies of the factors influencing the drivers’ subjective assessment of the ease of finding a vacant parking space in an area equipped with vehicle detection devices were conducted in Poland. The research questions reported by the authors allowed the characterization of drivers who positively assess the ease of finding a vacant parking space in an area equipped with vehicle detection devices. In addition, it is possible to reach a group of drivers who negatively assessed the ease of finding a vacant parking space to learn about the factors that may cause them to change the assessment to a positive one. The variables of the place of residence, the number of kilometers driven during a year, and the reason for choosing a parking space indicate groups of people who should be better informed about the operation of guidance systems for vacant parking spaces through information campaigns.

The results of the use of the parking space and the rotation indicator indicate the correct operation of the paid parking zone. The influence of the x_DPIBIGA_ variable in the presented logit model allows for the conclusion that dynamic parking information contributes to a driver’s positive subjective assessment of the ease of finding a vacant parking space in an area equipped with vehicle detection devices. Based on the constructed model, the use of a system for guiding vehicles to vacant parking spaces in areas with high demand for parking spaces is recommended for road managers (consisting of sensors detecting the presence of vehicles in parking spaces and dynamic parking information boards or mobile applications). The research results allow city authorities to better manage parking spaces equipped with vehicle detection devices in the paid parking zone. This may change the negative assessment of the ease of finding a vacant parking space into a positive one. Information about the use of parking spaces from sensors can be used for dynamic pricing for parking, as well as for the introduction of a parking reservation system.

In further research, it is planned to survey drivers who leave their vehicles in parking spaces equipped with devices detecting vehicles in the paid parking zone in other cities in Poland. Based on the newly obtained answers of the respondents, the model presented in this article will be verified and validated.

## Figures and Tables

**Table 1:** List of days on which a decrease in value is noticeable to the corresponding days in particular months of 2019, along with information about public holidays occurring on those days.

Month	Date	Day of the Week	Public Holiday
January	1, 5, and 6	Tuesday, Saturday, and Sunday	1—the New Year’s day 6—the Epiphany
February	-	-	-
March	-	-	-
April	19–22	Friday–Monday	21 and 22—Easter
May	1–5	Wednesday–Sunday	1—Labor Day 3—Constitution Day
June	20 and 21	Thursday and Friday	20—Corpus Christi
July	-	-	-
August	15 and 16	Thursday and Friday	15—Polish Army Day
September	-	-	-
October	-	-	-
November	1–3 and 11	Friday–Sunday and Monday	1—All Saints’ Day 11—National Independence Day
December	24–26 and 31	Tuesday–Thursday and Tuesday	24—Christmas Eve 25 and 26—Christmas 31—New Year’s Eve

**Table 2:** Characteristics of the respondents in terms of the ease of finding a vacant parking space in PPZ covered by DPI.

Question	Answer Options	Positive Assessment of the Ease of Finding a Vacant Parking Space in PPZ Covered by DPI	Neagtive Assessment of the Ease of Finding a Vacant Parking Space in PPZ Covered by DPI
Gender	Woman	57.78%	44.44%
	Man	42.22%	55.56%
Age	18–39	66.67%	62.50%
	39 and more	33.33%	37.50%
Place of residence	Gliwice and Gliwice county	40.00%	60.00%
	Other	60.00%	40.00%
Professional status	Working or student	75.56%	66.67%
	Other	24.44%	33.33%
Monthly income (net) (PLN)	0–3499	56.10%	62.50%
	3500 and more	43.90%	37.50%
Frequency of using the car as a driver	3–7 times a week	57.78%	77.78%
	Less often	42.22%	22.22%
The number of kilometers driven during a year	0–9999 km	52.27%	33.33%
	10,000 km and more	47.73%	66.67%
The number of years of having a driver’s license	1–9	42.22%	33.33%
	10 and more	57.78%	66.67%
The number of cars in the household	1	35.56%	50.00%
	2 and more	64.44%	50.00%
Trip purpose	Work, school or university	15.56%	22.22%
	Other	84.44%	77.78%
Frequency of using PPZ in Gliwice	1–7 times a week	40.00%	61.11%
	Less often	60.00%	38.89%
Subzone where respondents parked	Subzone A	77.78%	83.33%
	Subzone B	22.22%	16.67%
The reason for choosing a parking space	Close to the destination	86.67%	38.89%
	Low fee	13.33%	61.11%
Estimated parking time	0–2	67.65%	91.67%
	More than 2	32.35%	8.33%
Method of payment for parking	Payment at the parking meter	71.11%	72.22%
	Application on the phone	28.89%	27.78%
Guidance tools used to find a vacant parking space	Yes	55.56%	33.33%
	No	44.44%	66.67%
Subjective assessment of the search time for a vacant parking space	0–2	93.02%	33.33%
	More than 2	6.98%	66.67%
The number of passengers (apart from the driver) traveling by car	Yes	73.33%	77.78%
	No	26.67%	22.22%

**Table 3:** Coding for particular characteristics of variables.

No.	Variables	Characteristics	Coding
1	x_G_	Female	0
		Male	1
2	x_R_	Gliwice and Gliwice county	0
		Other	1
3	x_I_	0–3499 PLN	0
		3500 PLN and more	1
4	x_KC_	0–9999	0
		10,000 and more	1
5	x_FUPPZ_	1–7 times a week	0
		Less often	1
6	x_RCPS_	Close to the destination	0
		Low fee	1
7	x_DPIBIGA_	Dynamic parking information boards or ITS Gliwice application	0
		I do not use it	1
8	x_PTSPS_	0–2	0
		More than 2	1

**Table 4:** Results of the developed logit models.

Model Variables	1st Model 1		2nd Model	
	γ_i_	*p*-Value	γ_i_	*p*-Value
x_G_	−0.658	0.482	-	-
x_R_	−0.315	0.027	−0.124	0.021
x_I_	1.902	0.111	-	-
x_KC_	−0.479	0.032	−0.118	0.031
x_FUPPZ_	−0.322	0.021	−0.809	0.017
x_RCPS_	2.191	0.045	1.434	0.048
x_DPIBIGA_	−1.037	0.018	−0.644	0.015
x_PTSPS_	−3.218	0.002	−2.438	0.001
x_0_	−3.567	0.013	−2.188	0.014
% correctly predicted cases for the variable “No”	77.78 %		77.78 %	
% correctly predicted cases for the variable “Yes”	93.33 %		93.33 %	
R^2^ (N)	0.562		0.511	

## Data Availability

Not applicable.
